# Supplementary material for: Alterations in the Peritoneal Fluid Proteome of Horses with Colic Attributed to Ischemic and Non-Ischemic Intestinal Disease
Source: Animals (Basel). 2025 May 30;15(11):1604. doi: 10.3390/ani15111604 (PMC12153689; doi:10.3390/ani15111604)
Supplement: Supplementary file 1 [file animals-15-01604-s001.zip › animals-3627368-supplementary.pdf]

Alterations in the peritoneal fluid proteome of horses with intestinal disease

Rebecca C. Bishop[1], Justine V. Arrington[2], Pamela A. Wilkins[1], Annette McCoy[1]*

[1] Department of Veterinary Clinical Medicine, University of Illinois, 1008 W Hazelwood Dr, Urbana, Illinois
[2] Proteomics Core Facility, Roy J. Carver Biotechnology Center, University of Illinois, 1206 W Gregory Dr, Urbana, Illinois

**Table of Contents**	**Page**
Supplemental Table 1	S-2
Supplemental Table 2	S-4
Supplemental Table 3	S-8

Table S1. Peritoneal fluid proteins unique to groups of horses defined by lesion type (ischemic or non-ischemic) or by lesion location (large intestine or small intestine).

UniProtAC/ID	Protein Name	Gene Name
**Lesion type: Ischemic**		
A0A3Q2I8H8	Galectin 3 binding protein	LGALS3BP
A0A5F5PF14	Uncharacterized protein	BASP1
F7DYB1	Alpha-amylase	ENSECAP00000017078
O77811	Lactotransferrin	LTF
A0A5F5PJQ0	OS9 endoplasmic reticulum lectin	OS9
P05619	Leukocyte elastase inhibitor	SERPINB1
A0A3Q2LN10	Pentaxin	PTX3
A0A3Q2H2H2	Uncharacterized protein; Belongs to the peptidase S1 family	ENSECAP00000012520
F6Z230	Protein S100	S100A12
E1BDS9 *	Histone H3	H3C4
F6ZHQ5	Chloride intracellular channel protein	CLIC1
F6QB61	Peptidoglycan-recognition protein	ENSECAP00000015762
F6QKM4	Chymotrypsin C; Belongs to the peptidase S1 family.	CTRC
P68432 *	Tyrosine 3-monooxygenase/ tryptophan 5-monooxygenase activation protein gamma	YWHAG
A0A3Q2GVU8	Annexin	ANXA2
F6SG30	EF-hand domain-containing protein; Belongs to the S-100 family	ENSECAP00000029371
**Lesion type: Non-ischemic**		
P15437	Retinal dehydrogenase 1	ALDH1A1
H9GZQ2	Ig-like domain-containing protein	ENSECAP00000001751
A0A3Q2H5B8	Dihydropyrimidinase	DPYS
Q9XS63	p-Glu serpinin precursor	ENSECAP00000030274
F6XKT6	Transmembrane protein 120A	ENSECAP00000021835
F6ZRF6	Serpin family A member 7	SERPINA7
F6YS25	Carbonic anhydrase 3	CA2
F6PJ64	Major allergen Equ c 1	ENSECAP00000049682
F7DGD6	Melanotransferrin	MELTF
F6XI32	Ribosome biogenesis protein BOP1	BOP1
F7AHF3	Actin beta like 2	ACTBL2
**Lesion location: Large intestine**		
F7A6C3	Protocadherin 17	PCDH17
F6QKM4	Chymotrypsin C	CTRC
F6XI32	Ribosome biogenesis protein BOP1	BOP1

Q9XS63	p-Glu serpinin precursor	ENSECAP00000030274
A0A5F5PJQ0	Endoplasmic reticulum lectin 1	ERLEC1
F7AHF3	Actin beta like 2	ACTBL2
F7DGD6	Melanotransferrin	MELTF
F6XKT6	Transmembrane protein 120A	ENSECAP00000021835
**Lesion location: Small intestine**		
A0A3Q2LN10	Pentaxin	PTX3
A0A3Q2I8H8	Galectin 3 binding protein	LGALS3BP
F6SG30	EF-hand domain-containing protein: Belongs to the S-100 family	ENSECAP00000029371
F6PJ64	Major allergen Equ c 1	ENSECAP00000049682
F6QB61	Peptidoglycan-recognition protein	ENSECAP00000015762
A0A3Q2H5B8	Dihydropyrimidinase	DPYS
P05619	Leukocyte elastase inhibitor	SERPINB1
F6Z230	Protein S100	S100A12
P15437	Retinal dehydrogenase 1	ALDH1A1
F7D635	Uncharacterized protein	ZBED4
F6RP73	Uncharacterized protein	ENSECAP00000022058

Table S2. Results of differential abundance analysis of 129 proteins found in the peritoneal fluid of 20 horses with colic, comparing between groups based on lesion type (ischemic vs non-ischemic) and lesion location (large vs small intestine). Proteins are sorted in descending order of fold change (Log_2FC) for each comparison. Proteins with a p value < 0.05 (highlighted in yellow) were considered of interest; there were no significantly DE proteins based on FDR <0.05.

**Ischemic (-) vs Non-ischemic (+)**				**Large intestine (-) vs Small intestine (+)**			
**Uniprot AC/ID**	**Log_2FC**	**p value**	**FDR**	**Uniprot AC/ID**	**Log_2FC**	**p value**	**FDR**
A0A5F5PYW9	1.75	0.537	0.999	A0A3Q2H875	5.07	0.055	0.713
A0A3Q2GWV7	0.76	0.793	0.999	O46375	3.23	0.262	0.988
F6RRV1	0.75	0.034*	0.396	P02062	2.4	0.018*	0.426
F6R5Y2	0.7	0.604	0.999	F6VBP9	2.15	0.355	0.988
A0A3Q2LBP6	0.65	0.002*	0.209	A0A3Q2HW24	1.85	0.402	0.988
A0A3Q2GU85	0.65	0.821	0.999	P01958	1.83	0.023*	0.426
P14452	0.6	0.75	0.999	A0A3Q2GX59	1.76	0.542	0.988
F7BQD6	0.56	0.433	0.999	F7BXD8	1.65	0.395	0.988
Q28369	0.51	0.468	0.999	A0A3Q2KXN9	1.62	0.481	0.988
A0A5F5PLV2	0.42	0.664	0.999	P14452	1.51	0.415	0.988
F6ZI35	0.38	0.035	0.396	A0A3Q2HRX3	1.5	0.001*	0.085
A0A3Q2I5T2	0.37	0.899	0.999	P0DN28	1.34	0.388	0.988
A0A3Q2HMJ3	0.36	0.153	0.756	A0A5F5PUU0	1.15	0.63	0.988
F6ZLR1	0.35	0.18	0.798	A0A3Q2I2H1	1.07	0.71	0.988
F6W2Y1	0.34	0.682	0.999	F6R1X9	1.03	0.641	0.988
A0A3Q2LE47	0.33	0.027	0.392	F7BTW7	1	0.396	0.988
F7DZE7	0.33	0.878	0.999	A0A5F5PYW9	0.98	0.732	0.988
F7CYR1	0.31	0.016	0.316	H9GZN9	0.97	0.016*	0.426
F6Z129	0.31	0.792	0.999	F7CFN5	0.97	0.738	0.988
F7AW05	0.27	0.052	0.475	F7A1W7	0.91	0.003*	0.208
A0A3Q2IDE0	0.27	0.685	0.999	F6QFD9	0.82	0.593	0.988
F7BKE1	0.25	0.177	0.798	A0A5F5PST7	0.72	0.803	0.988
A0A5F5PJQ9	0.25	0.781	0.999	F6PUW3	0.71	0.645	0.988
A0A3Q2HTG2	0.24	0.693	0.999	F6W2Y1	0.65	0.437	0.988
F7DRS2	0.23	0.742	0.999	A0A5F5PJQ9	0.61	0.494	0.988
F6RZ27	0.22	0.344	0.999	A0A3Q2HTG2	0.59	0.323	0.988
F7CSL8	0.22	0.038	0.396	A0A5F5PGW4	0.54	0.221	0.988
F7C450	0.21	0.083	0.535	P83704	0.5	0.096	0.797
A0A3Q2H333	0.19	0.068	0.484	A0A5F5Q1N6	0.5	0.685	0.988
F6USP9	0.18	0.015	0.316	Q28369	0.44	0.532	0.988
F6XWM5	0.18	0.585	0.999	A0A3Q2I8Y6	0.44	0.006	0.262
F6QYS3	0.16	0.811	0.999	H9GZV1	0.44	0.198	0.988

A0A5F5PUE2	0.16	0.126	0.707	F6RI47	0.43	0.09	0.797
F6W4R2	0.15	0.38	0.999	F7DU87	0.42	0.252	0.988
F7CN11	0.14	0.651	0.999	F7AXF6	0.38	0.896	0.988
A0A3Q2HWQ6	0.14	0.295	0.999	A0A3Q2KTQ9	0.36	0.84	0.988
F7BM31	0.13	0.253	0.999	A0A5F5PIE1	0.36	0.891	0.988
A0A3Q2H846	0.12	0.539	0.999	F6Z5L1	0.36	0.88	0.988
A0A3Q2I427	0.11	0.481	0.999	F7CZW9	0.32	0.036	0.577
H9GZQ9	0.1	0.632	0.999	F7CN11	0.3	0.334	0.988
Q28372	0.09	0.562	0.999	F7DZE7	0.29	0.893	0.988
F7BTW7	0.08	0.945	0.999	F6REX3	0.29	0.903	0.988
F6UN85	0.07	0.759	0.999	F7CAC5	0.27	0.424	0.988
A0A5F5PW18	0.07	0.294	0.999	F6R5Y2	0.25	0.855	0.988
F7BFJ1	0.06	0.27	0.999	A0A3Q2HVB6	0.24	0.28	0.988
H9GZT5	0.05	0.737	0.999	A0A5F5PXB8	0.24	0.737	0.988
F7B3I5	0.03	0.76	0.999	H9GZV0	0.24	0.935	0.988
F6T0P6	0.03	0.783	0.999	A0A3Q2HMJ3	0.22	0.391	0.988
A0A3Q2KNA7	0.03	0.965	0.999	A0A5F5PU06	0.2	0.218	0.988
F6UL68	0.02	0.907	0.999	F6XSF7	0.2	0.174	0.988
A0A5F5PEG5	0.02	0.847	0.999	F6T7X3	0.19	0.784	0.988
A0A5F5Q326	0.01	0.908	0.999	F6SUS2	0.18	0.792	0.988
F7DGR1	0.01	0.95	0.999	A0A3Q2GXU9	0.16	0.499	0.988
F6QDQ1	0	0.999	0.999	F6W4R2	0.16	0.364	0.988
P38029	0	0.996	0.999	H9GZU8	0.15	0.722	0.988
A0A3Q2HVB6	-0.01	0.977	0.999	F7B3I5	0.14	0.183	0.988
F7APU2	-0.02	0.867	0.999	F6X1I8	0.14	0.375	0.988
F6Z2L5	-0.02	0.83	0.999	A0A3Q2IDE0	0.12	0.858	0.988
F7DXM5	-0.03	0.872	0.999	F6T0P6	0.12	0.28	0.988
A0A3Q2H7N4	-0.04	0.892	0.999	F6PKE1	0.11	0.331	0.988
A0A5F5PUU0	-0.04	0.985	0.999	F7DTV1	0.11	0.876	0.988
A0A3Q2IDD2	-0.06	0.529	0.999	F7BQD6	0.11	0.881	0.988
A0A3Q2L2R4	-0.06	0.933	0.999	A0A3Q2L2R4	0.09	0.898	0.988
A0A3Q2GWN9	-0.07	0.438	0.999	F6QYS3	0.09	0.895	0.988
F7ASE1	-0.07	0.96	0.999	F7E3H8	0.08	0.672	0.988
A0A3Q2L3I3	-0.08	0.726	0.999	F6QX36	0.07	0.537	0.988
A0A3Q2I8Y6	-0.08	0.684	0.999	A0A3Q2L3I3	0.07	0.762	0.988
A0A3Q2KTQ9	-0.08	0.965	0.999	H9GZQ9	0.06	0.778	0.988
F6PKE1	-0.08	0.476	0.999	A0A5F5PW18	0.05	0.432	0.988
P48770	-0.09	0.928	0.999	A0A5F5PM55	0.05	0.688	0.988
F6R1X9	-0.09	0.968	0.999	F6Z041	0.04	0.731	0.988
F7CZW9	-0.09	0.591	0.999	A0A3Q2GWN9	0.04	0.625	0.988
F6Z041	-0.11	0.354	0.999	F6Z2L5	0	0.964	0.988

A0A3Q2HJE2	-0.12	0.958	0.999	F7BFJ1	0	0.974	0.988
F7E3H8	-0.12	0.539	0.999	F6PQ46	0	0.988	0.988
F6T7X3	-0.13	0.858	0.999	F6VJR6	-0.02	0.937	0.988
F7A692	-0.13	0.449	0.999	F6Y0C1	-0.02	0.983	0.988
F6X1I8	-0.13	0.39	0.999	H9GZT5	-0.03	0.849	0.988
F6SUS2	-0.16	0.816	0.999	F6UL68	-0.03	0.814	0.988
A0A5F5PM55	-0.16	0.158	0.756	A0A3Q2LE47	-0.05	0.783	0.988
F6VJR6	-0.17	0.534	0.999	A0A3Q2HWQ6	-0.05	0.723	0.988
A0A3Q2HD35	-0.18	0.331	0.999	A0A3Q2HD35	-0.05	0.786	0.988
F6PQ46	-0.18	0.186	0.798	F6RZ27	-0.06	0.807	0.988
F6QX36	-0.19	0.064	0.484	A0A5F5PLV2	-0.06	0.95	0.988
A0A5F5PYP6	-0.19	0.947	0.999	F6V881	-0.07	0.738	0.988
F6V881	-0.24	0.261	0.999	F7APU2	-0.07	0.435	0.988
Q28380	-0.26	0.92	0.999	F7DXM5	-0.08	0.7	0.988
A0A5F5PU06	-0.29	0.058	0.483	A0A5F5PWX5	-0.08	0.972	0.988
F7DTV1	-0.31	0.662	0.999	F7CSL8	-0.09	0.459	0.988
F6Z5L1	-0.33	0.89	0.999	A0A3Q2IDD2	-0.09	0.323	0.988
F6XSF7	-0.33	0.012	0.316	A0A5F5PEG5	-0.1	0.237	0.988
F6REX3	-0.36	0.88	0.999	F7C450	-0.1	0.421	0.988
A0A3Q2GXU9	-0.39	0.073	0.497	A0A5F5Q326	-0.11	0.318	0.988
A0A5F5PXB8	-0.41	0.557	0.999	F7DGR1	-0.11	0.312	0.988
F6VBP9	-0.44	0.854	0.999	F6USP9	-0.11	0.19	0.988
F7DU87	-0.44	0.221	0.92	F7A692	-0.11	0.514	0.988
P83704	-0.45	0.139	0.748	F7BM31	-0.12	0.297	0.988
A0A5F5PGW4	-0.47	0.291	0.999	A0A3Q2H333	-0.13	0.25	0.988
P0DN28	-0.49	0.753	0.999	A0A5F5PUE2	-0.13	0.233	0.988
A0A3Q2KXN9	-0.5	0.83	0.999	F6ZLR1	-0.13	0.624	0.988
A0A5F5PST7	-0.5	0.861	0.999	Q28372	-0.16	0.297	0.988
F7A1W7	-0.51	0.149	0.756	F7DRS2	-0.17	0.809	0.988
H9GZV0	-0.54	0.852	0.999	P38029	-0.17	0.826	0.988
F6RI47	-0.64	0.005	0.316	F6ZRH8	-0.18	0.951	0.988
F6Y0C1	-0.64	0.58	0.999	A0A3Q2H846	-0.18	0.359	0.988
H9GZU8	-0.66	0.101	0.621	A0A3Q2HJE2	-0.18	0.934	0.988
H9GZV1	-0.66	0.04*	0.396	A0A3Q2LBP6	-0.23	0.357	0.988
F7CAC5	-0.79	0.008*	0.316	A0A3Q2I427	-0.24	0.097	0.797
F7AXF6	-0.84	0.769	0.999	F7CYR1	-0.25	0.064	0.751
P02062	-0.86	0.429	0.999	A0A3Q2GU85	-0.29	0.919	0.988
P27425	-0.91	0.536	0.999	F6ZI35	-0.31	0.098	0.797
F6PUW3	-0.92	0.551	0.999	F7AW05	-0.31	0.022	0.426
H9GZN9	-0.95	0.018	0.316	A0A3Q2H7N4	-0.32	0.244	0.988
F7CFN5	-0.97	0.738	0.999	F7BKE1	-0.35	0.047	0.674

F7BF31	-1.02	0.708	0.999	A0A3Q2KNA7	-0.35	0.603	0.988
F7BXD8	-1.02	0.604	0.999	F6RRV1	-0.36	0.343	0.988
A0A5F5Q1N6	-1.1	0.364	0.999	F6UN85	-0.37	0.099	0.797
F6QFD9	-1.13	0.456	0.999	F6QDQ1	-0.38	0.805	0.988
A0A3Q2HRX3	-1.14	0.02*	0.316	F7BF31	-0.42	0.879	0.988
P01958	-1.31	0.118	0.692	H9GZR2	-0.42	0.619	0.988
A0A3Q2I2H1	-1.34	0.641	0.999	F6XWM5	-0.43	0.175	0.988
H9GZR2	-1.51	0.06	0.483	F7ASE1	-0.51	0.71	0.988
A0A3Q2HW24	-1.64	0.458	0.999	P48770	-0.54	0.574	0.988
A0A3Q2GX59	-1.77	0.539	0.999	A0A5F5PYP6	-0.64	0.825	0.988
A0A5F5PIE1	-1.9	0.458	0.999	F6Z129	-0.81	0.484	0.988
A0A5F5PWX5	-1.93	0.376	0.999	A0A3Q2I5T2	-0.94	0.744	0.988
F6ZRH8	-2.19	0.447	0.999	P27425	-0.98	0.505	0.988
A0A3Q2H875	-2.21	0.41	0.999	A0A3Q2GWV7	-1.07	0.712	0.988
O46375	-2.3	0.425	0.999	Q28380	-1.37	0.595	0.988

Table S3. Functional enrichment analysis results from STRING v.11.5 for the 61 proteins upregulated in the peritoneal fluid (PF) of horses with ischemic intestinal lesions, and the 59 proteins upregulating in PF of horses with small intestinal lesions. Inclusion criteria for enriched functions was FDR < 0.05 and enrichment strength ≥1. Gene count reflects the number of proteins in the network (Obs) that are annotated with a given term, compared to the number in the background (Bgnd).

Term ID	Description	Gene Count		Strength	FDR
		Obs	Bgnd		
**Biological Process (Gene Ontology)**					
GO:0006956	Complement activation	18	85	1.86	7.05E-24
GO:0006959	Humoral immune response	20	220	1.49	1.51E-20
GO:0006958	Complement activation, classical pathway	14	46	2.02	3.58E-20
GO:0016064	Immunoglobulin mediated immune response	15	100	1.71	5.97E-18
GO:0002252	Immune effector process	22	483	1.19	2.31E-17
GO:0002443	Leukocyte mediated immunity	17	213	1.44	1.13E-16
GO:0006957	Complement activation, alternative pathway	9	19	2.21	4.89E-14
GO:0050776	Regulation of immune response	20	600	1.06	3.04E-13
GO:0002250	Adaptive immune response	16	349	1.2	3.50E-12
GO:0097006	Regulation of plasma lipoprotein particle levels	6	37	1.75	1.04E-06
GO:0030301	Cholesterol transport	6	38	1.73	1.16E-06
GO:0033344	Cholesterol efflux	5	18	1.98	2.09E-06
GO:0051346	Negative regulation of hydrolase activity	11	368	1.01	3.61E-06
GO:0071827	Plasma lipoprotein particle organization	5	27	1.8	1.02E-05
GO:0034381	Plasma lipoprotein particle clearance	4	15	1.96	6.54E-05
GO:0034369	Plasma lipoprotein particle remodeling	4	19	1.86	0.00014
GO:0034382	Chylomicron remnant clearance	3	4	2.41	0.00019
GO:0034380	High-density lipoprotein particle assembly	3	7	2.17	0.00057
GO:0033700	Phospholipid efflux	3	8	2.11	0.00076
GO:0042157	Lipoprotein metabolic process	5	105	1.21	0.0031
GO:0030449	Regulation of complement activation	3	15	1.84	0.0033
GO:1905952	Regulation of lipid localization	5	108	1.2	0.0033
GO:0055088	Lipid homeostasis	5	120	1.15	0.0051
GO:0010916	Negative regulation of very-low-density lipoprotein particle clearance	2	3	2.36	0.0127
GO:1905907	Negative regulation of amyloid fibril formation	2	3	2.36	0.0127
GO:0006910	Phagocytosis, recognition	3	27	1.58	0.013
GO:0090207	Regulation of triglyceride metabolic process	3	33	1.49	0.0207
GO:0032374	Regulation of cholesterol transport	3	40	1.41	0.033
GO:0007596	Blood coagulation	4	108	1.1	0.0386
GO:0032375	Negative regulation of cholesterol transport	2	8	1.93	0.0433

GO:0034374	Low-density lipoprotein particle remodeling	2	8	1.93	0.0433
GO:0050853	B cell receptor signaling pathway	3	47	1.34	0.0473
**Molecular function (Gene Ontology)**					
GO:0004857	Enzyme inhibitor activity	12	338	1.09	7.71E-07
GO:0034987	Immunoglobulin receptor binding	6	24	1.93	7.71E-07
GO:0004866	Endopeptidase inhibitor activity	9	168	1.26	2.05E-06
GO:0070325	Lipoprotein particle receptor binding	5	21	1.91	4.60E-06
GO:0003823	Antigen binding	7	97	1.39	7.67E-06
GO:0004867	Serine-type endopeptidase inhibitor activity	7	104	1.36	1.07E-05
GO:0070653	High-density lipoprotein particle receptor binding	3	3	2.54	0.00011
GO:0008201	Heparin binding	7	168	1.15	0.00017
GO:0005344	Oxygen carrier activity	3	9	2.06	0.00098
GO:0019825	Oxygen binding	3	13	1.9	0.0022
GO:0055102	Lipase inhibitor activity	3	14	1.87	0.0025
**Cellular Component (Gene Ontology)**					
GO:0005615	Extracellular space	40	1384	1	2.54E-29
GO:0034364	High-density lipoprotein particle	9	28	2.04	4.72E-13
GO:0042627	Chylomicron	6	11	2.27	1.22E-09
GO:0034361	Very-low-density lipoprotein particle	6	14	2.17	3.33E-09
GO:0034363	Intermediate-density lipoprotein particle	4	4	2.54	6.78E-07
GO:0034366	Spherical high-density lipoprotein particle	4	5	2.44	1.11E-06
GO:0005579	Membrane attack complex	4	6	2.36	1.69E-06
GO:0019814	Immunoglobulin complex	5	32	1.73	8.29E-06
GO:0005833	Hemoglobin complex	3	6	2.23	0.0002
GO:0034362	Low-density lipoprotein particle	3	11	1.97	0.00079
**Local network clusters (STRING)**					
CL:17289	Complement and coagulation cascades, and lipoprotein particle	29	141	1.85	1.45E-41
CL:17290	Protein-lipid complex, and Complement and coagulation cascades	16	98	1.75	2.88E-20
CL:17292	Mixed, incl. lipoprotein particle, and negative regulation of fibrinolysis	14	59	1.91	1.50E-19
CL:17488	Complement activation, classical pathway, and complement activation, lectin pathway	11	40	1.97	1.35E-15
CL:17489	Complement activation, classical pathway, and complement activation, lectin pathway	9	26	2.07	2.99E-13
CL:17294	Mixed, incl. negative regulation of fibrinolysis, and fatty acid binding	9	29	2.03	6.09E-13
CL:17297	Mixed, incl. negative regulation of fibrinolysis, and high-density lipoprotein particle receptor binding	8	23	2.08	1.06E-11

CL:15855	B cell receptor signaling pathway, and immunoglobulin production	8	51	1.73	2.55E-09
CL:15856	B cell receptor signaling pathway, and B cell receptor signaling pathway	7	39	1.79	2.25E-08
CL:17298	Mixed, incl. negative regulation of fibrinolysis, and high-density lipoprotein particle receptor binding	6	18	2.06	2.25E-08
CL:17369	Intermediate-density lipoprotein particle, and regulation of very-low-density lipoprotein particle remodeling	4	6	2.36	3.33E-06
CL:17354	Lipoprotein particle	5	27	1.8	7.84E-06
CL:15857	B cell receptor signaling pathway, and B cell receptor signaling pathway	5	33	1.72	1.75E-05
CL:17300	Mixed, incl. high-density lipoprotein particle receptor binding, and protein activation cascade	4	12	2.06	2.23E-05
CL:17526	Membrane attack complex	3	5	2.31	0.00021
CL:18146	Hemoglobin complex, and hemoglobin binding	3	6	2.23	0.0003
CL:18033	Mixed, incl. iron ion transport, and gas transport	5	75	1.36	0.00053
CL:17495	Complement activation, lectin pathway, and synapse pruning	3	10	2.01	0.00094
CL:15894	Mixed, incl. phagocytosis, recognition, and IgA binding	3	13	1.9	0.0018
CL:15923	IgG binding, and immunoglobulin receptor binding	2	5	2.14	0.0215
CL:17496	Synapse pruning, and negative regulation of complement activation	2	5	2.14	0.0215
CL:17543	C5a anaphylatoxin chemotactic receptor binding, and inner acrosomal membrane	2	6	2.06	0.0264
**KEGG**					
ecb04610	Complement and coagulation cascades	20	91	1.88	8.94E-29
ecb04979	Cholesterol metabolism	7	48	1.7	3.93E-08
ecb05322	Systemic lupus erythematosus	10	223	1.19	1.51E-07
ecb05143	African trypanosomiasis	6	66	1.49	5.54E-06
ecb05150	Staphylococcus aureus infection	8	187	1.17	6.54E-06
ecb05133	Pertussis	6	77	1.43	8.70E-06
ecb05146	Amoebiasis	7	141	1.23	1.20E-05
ecb05340	Primary immunodeficiency	3	61	1.23	0.0334
**Subcellular localization (COMPARTMENTS)**					
GOCC:0005576	Extracellular region	48	1549	1.03	4.80E-39
GOCC:0005615	Extracellular space	29	507	1.29	2.86E-27
GOCC:0034364	High-density lipoprotein particle	9	27	2.06	4.29E-13
GOCC:0042627	Chylomicron	7	13	2.27	2.40E-11
GOCC:0034361	Very-low-density lipoprotein particle	7	18	2.12	1.29E-10
GOCC:0034366	Spherical high-density lipoprotein particle	6	8	2.41	2.51E-10

GOCC:0005579	Membrane attack complex	6	9	2.36	3.79E-10
GOCC:0042571	Immunoglobulin complex, circulating	6	14	2.17	2.66E-09
GOCC:0034363	Intermediate-density lipoprotein particle	5	6	2.46	1.12E-08
GOCC:0019814	Immunoglobulin complex	7	47	1.71	2.54E-08
GOCC:0034362	Low-density lipoprotein particle	5	14	2.09	2.24E-07
GOCC:0031838	Haptoglobin-hemoglobin complex	3	7	2.17	0.00028
GOCC:0005833	Hemoglobin complex	3	12	1.93	0.00094
GOCC:0034360	Chylomicron remnant	2	3	2.36	0.0071
GOCC:0062167	Complement component C1q complex	2	3	2.36	0.0071
GOCC:0062136	Low-density lipoprotein receptor complex	2	5	2.14	0.0136
**UniProt Keywords**					
KW-0768	Sushi	6	58	1.55	3.78E-06
KW-0393	Immunoglobulin domain	6	96	1.33	4.41E-05
KW-0561	Oxygen transport	3	9	2.06	0.00035
KW-0349	Heme	4	109	1.1	0.019
